# The Efficacy and Predictors of Using GPi-DBS to Treat Early-Onset Dystonia: An Individual Patient Analysis

**DOI:** 10.1155/2021/9924639

**Published:** 2021-05-07

**Authors:** Wenxiu Chen, Houyou Fan, Guohui Lu

**Affiliations:** ^1^Department of Neurosurgery, The First Affiliated Hospital of Nanchang University, Nanchang, Jiangxi, China; ^2^The First Clinical Medical College of Nanchang University, Nanchang, Jiangxi, China

## Abstract

**Objective:**

To compare the efficacy in patients with different genotypes, identify the potential predictive factors, and summarize the complications of globus pallidus deep brain stimulation (GPi-DBS) treating early-onset dystonia.

**Methods:**

Three electronic databases (PubMed, Embase, and Cochrane databases) were searched with no publication data restriction. The primary outcomes were the improvements in Burke–Fahn–Marsden Dystonia Rating Scale motor (BFMDRS-M) and disability (BFMDRS-D) score. Pearson's correlation coefficients and a metaregression analysis were used to identify the potential predictive factors. This article was registered in Prospero (CRD42020188527).

**Results:**

Fifty-four studies (231 patients) were included. Patients showed significant improvement rate in BFMDRS-M (60.6%, *p* < 0.001) and BFMDRS-D (57.5%, *p* < 0.001) scores after treatment with GPi-DBS. BFMDRS-M score improved greater in the DYT-1-positive (*p* = 0.001) and DYT-11-positive (*p* = 0.008) patients compared to DYT-6-positive patients. BFMDRS-D score improved greater in the DYT-11 (+) compared to DYT-6 (+) patients (*p* = 0.010). The relative change of BFMDRS-M (*p* = 0.002) and BFMDRS-D (*p* = 0.010) scores was negatively correlated with preoperative BFMDRS-M score. In the metaregression analysis, the best predictive model showed that preoperative BFMDRS-M, disease duration (*p* = 0.047), and the age at symptom onset (*p* = 0.027) were important.

**Conclusion:**

Patients with early-onset dystonia have a significant effect after GPi-DBS treatment, and DYT-1 (+) and DYT-11 (+) patients are better candidates for GPi-DBS. Lower preoperative score, later age of onset, and an earlier age at surgery probably predict better clinical outcomes.

## 1. Background

Early-onset dystonia is one type of dystonia, characterized by onset age ≤ 26, often presents with the onset of lower or upper limb symptoms, and progresses to other parts of the body [1]. According to the existing literature, early-onset dystonia is highly correlated with genetic mutations, mainly including DYT-1, DYT-6, DYT-11, and DYT-28 [2].

DYT-1 (+) patients present onset symptoms in childhood or adolescence, typically with dystonia of a single limb that often spreads within 1-3 years to involve all four limbs [3, 4]. The onset age of DYT-6 (+) patients is also during childhood and adolescence, but more than half of patients started onset symptoms earlier than adolescence. Approximately 25% of DYT-6 (+) patients present with cervical dystonia [5]. Patients with DYT-11 genotype present with myoclonus, dystonia, or both, but most patients present with myoclonus in childhood or adolescence [6, 7]. The patients with DYT-28 (+) are registered as an early-onset generalized dystonia with age at onset of symptoms generally between 4 and 6 years old [8].

Clinically, many methods are used to treat dystonia, such as drug therapy, endotoxin injection, and thalamotomy [9, 10]. But for chronic and refractory dystonia, deep brain stimulation (DBS) is the most effective and safe method [11, 12]. Many researches have shown that the globus pallidus DBS (GPi-DBS) is effective for DYT-1 (+) [13] and DYT-11 (+) early-onset dystonia [14]. But for DYT-6 (+) and DYT-28 (+), further research is still needed to identify the efficacy of GPi-DBS [15, 16]. What is more, no literature has reported on whether there are differences in the efficacy of GPi-DBS treating different genotypes of early-onset dystonia as well as the rate of response and complications. The surgery indications and predictive factors of GPi-DBS treating early-onset dystonia are also not clearly stated in the clinical guidelines.

Therefore, we conducted an individual patient data meta-analysis of all published studies to explore the efficacy, predictors, and complications of GPi-DBS treating early-onset dystonia.

## 2. Methods

### 2.1. Search Strategy

The Preferred Reporting Items for Systematic Reviews and Meta-Analysis (PRISMA) guidelines were followed when we were searching literatures. The keywords “early-onset dystonia,” “child-onset dystonia,” “childhood onset dystonia,” “DYT-TOR1A,” “DYT-1,” “DYT-THAP1,” “DYT-6,” “DYT-SGCE,” “DYT-11,” “DYT-KMT2B,” “DYT-28,” and “deep brain stimulation” were searched in the PubMed, Embase, and Cochrane Central Register of Controlled Trials and Cochrane Movement Disorders Group Trials Register. Two reviewers (CWX and FHY) independently scanned the titles and abstracts and then reviewed the full texts.

### 2.2. Inclusion and Exclusion Criteria

The inclusion criteria for publications were as follows: (1) baseline characteristics of patients (gender, age at symptom onset, operating age, basic dystonia movement score, and disability); (2) surgical operation (DBS target, stimulation settings, and microelectrode recording (MER)); and (3) outcomes (motor and disability scores of the Burke–Fahn–Marsden Dystonia Rating Scale (BFMDRS-M, BFMDRS-D), follow-up time, and complications mentioned in the article). The collated data are listed in Supplementary Table S1. The published evidence was evaluated following the Oxford Centre for Evidence-based Medicine (OCEBM) Levels of Evidence [17].

Studies were excluded for the following reasons: (1) other surgeries, like thalamotomy, were conducted; (2) other nuclei were targeted; (3) patients have been reported elsewhere; (4) studies were conference articles, letters, or editions.

### 2.3. Data Extraction

Available data of all patients were extracted. All randomized clinical trials, case series, case reports, and case-control studies that reported demographics, surgery, and outcome data of patients with early-onset dystonia who underwent GPi-DBS surgery were taken into consideration. There were no restrictions on the release date, release status, and language. We searched Medline and browsed reference lists of articles and assessed the research based on these articles. The year of publication, author's name, demographics, and result data were juxtaposed to exclude duplicate patients. Therefore, the data extraction table was gradually developed, piloted, and improved.

### 2.4. Genotyping

Early-onset dystonia is generally associated with one single genetic mutation, including DYT-1, DYT-6, DYT-11, and DYT-28. DYT-11 (+) and DYT-28 (+) dystonias are both syndromes, which are considered combining dystonia with additional symptoms. DYT-11 (+) is accompanied by myoclonus, while DYT-28 (+) is accompanied by intellectual disability and infantile deformity. Due to insufficient data, DYT-28 (+) was excluded in the comparisons. When the patient's genetic confirmation was not mentioned, only patients with specific diagnosis of dystonia and whose age of onset meets the criteria were included in the early onset of unknown dystonia.

### 2.5. Analysis Strategy

In order to perform a statistical analysis of the results of the severity of dystonia, the effective changes in the BFMDRS-M and BFMDRS-D were included. Values were extracted using Get Data Graph Digitizer when data were displayed only by graph. The data of demographic characteristics and basic dystonia score are all sorted out and got two characteristic statistical indicators: mean and median. In the BFMDRS scoring system, higher scores indicate more severe diseases, and the relative change rates are calculated with the following algorithm: preoperative scores are divided by the difference between preoperative and postoperative scores [18].

### 2.6. Statistical Analysis

A paired Student *t* test was performed to compare baseline (0 months) with the score in different time categories after surgery (0 to ≤6, >6 to ≤12, >12 to ≤24, >24 to ≤36, and >36 months). The relative changes in postoperative BFMDRS-M and BFMDRS-D versus baseline data represented the primary results. We compared the relative change rates between different genotypes of early-onset dystonia using two independent-samples *t* test [19]. To determine potential predictive factors, Pearson's correlation coefficients and a stepwise multivariate regression analysis were performed between the relative change rates of BFMDRS-M or BFMDRS-D and different predictive factors, including symptom onset (years), course of disease (years), age of surgery (years), MER, follow-up period (months), and preoperative BFMDRS score. Secondary outcomes were the complication rate. Furthermore, since most of the patients were followed up under 3 years, a restriction on the follow-up time of 3 years was set up. Then, we reperformed the same statistical analyses. The statistically significant definition standard is *p* ≤ 0.05 [20]. We used SPSS 25.0 (IBM, Armonk, NY) to calculate statistics. When no obvious abnormality was found, Student's *t* test was used after the comprehensive normality test of D'Agostino and Pearson [21].

## 3. Results

### 3.1. Search Results

**Figure 1:**
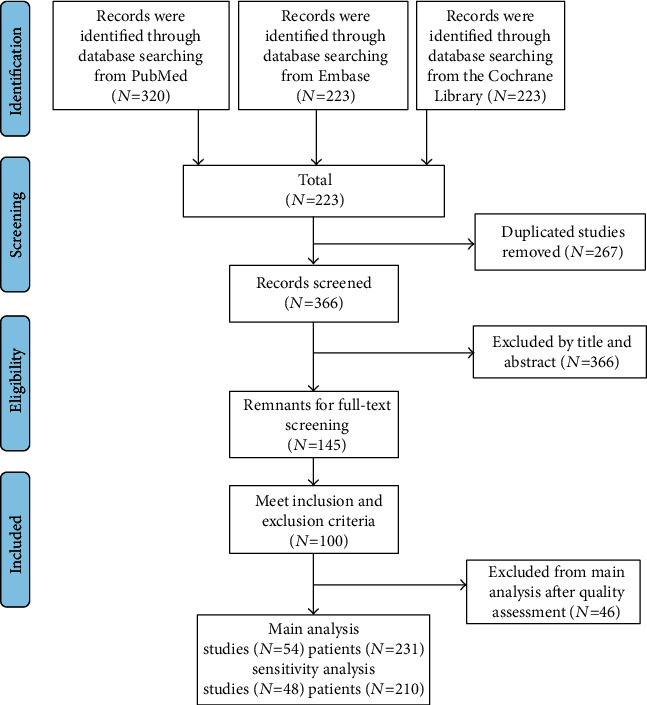
Flow diagram based on PRISMA statement (https://www.prisma-statement.org).

Among the 366 studies, 54 studies were screened out from the inclusion and exclusion criteria, and the data were extracted (Figure 1). A sample size of 231 patients was distributed into DYT-1 (*n* = 101), DYT-6 (*n* = 27), and DYT-11 (*n* = 32) mutations. Specific documents and data are placed in Supplementary Table S1. All the 54 studies were qualified as level 4 evidence based on the OCEBM.

### 3.2. Demographics

DYT-1 (+) patients (*n* = 101) largely outnumbered DYT-6 (+) (*n* = 27) and DYT-11 (+) (*n* = 32) patients. The dystonia genotype could not be determined for 71 patients.  Table 1 summarizes the demographic characteristics and basic dystonia score. The mean (range) age at onset of all the 231 patients (110 males, 98 females, and 26 unknown) was 8.9 (0–25) years. Their age at surgery was 24.3 (5.3–72) years, with a disease duration of 15.4 (0.5–66) years. There were no significant differences in the composition of gender, age at symptom onset, age at surgery, and disease duration between DYT-1 (+) and DYT-6 (+). The results of this statistical analysis were the same between DYT-11 (+) and DYT-6 (+), while there existed differences in the duration of the disease. As for DYT-11 (+) and DYT-1 (+), there were no significant differences in the composition of gender and age at symptom onset, but the age at surgery and disease duration between them were of obvious difference.

Baseline data for the three-year follow-up period are shown in Table 2. The disease duration among DYT-6 (+) and DYT-11 (+) was of no significant difference in the three-year follow-up. All the other results of baseline during the three-year follow-up were the same as those at the unrestricted time.

### 3.3. Surgery Programming and Microelectrode Recordings

All patients underwent bilateral surgery, and all the DBS targets of the extracted article data were GPi. 120 patients used MER. In general, the GPi-DBS amplitudes on the right ranged from 0.5 to 5.6 V (mean of 2.8 V), and the GPi-DBS amplitudes on the left ranged from 0.5 to 4.5 V (mean of 2.7 V) with a frequency bilaterally ranging between 60 and 451 Hz (median of 124.5 Hz). The mean programmed pulse width for the right and left was 155.4 microseconds and 152.4 microseconds, respectively. The bilateral stimulus parameters of DYT-6 (+) were consistent, and all the bilateral frequency stimuli parameters of the early-onset muscle tension disorder were consistent. Half of the patients used microelectrodes.

### 3.4. The BFMDRS-M and BFMDRS-D Changes at the Five Follow-Up Times

The median follow-up time reported after GPi-DBS was 12 months (1-196 months), and the length of follow-up for patients in the DYT-6 (+) group was longer than that for patients in the DYT-11 (+) group (2-196 months vs. 1-20 months, respectively; *p* = 0.003). The length of follow-up for the patients in the DYT-6 (+) group was longer than that for the patients in DYT-1 (+) group (2-196 months vs. 1-102 months, respectively; *p* = 0.01). Considering the follow-up times showed obvious heterogeneity, the BFMDRS-M and BFMDRS-D were divided into five follow-up time groups as follows: 0 to ≤6, >6 to ≤12, >12 to ≤24, >24 to ≤36, and >36 months.  Table 3 shows that, during the 0- to ≤6-, >6- to ≤12-, >12- to ≤24-, >24- to ≤36-, and >36-month time categories, the BFMDRS-M scores were 23.6 ± 24.9 (0–94.5, *n* = 97), 17.1 ± 17.9 (0–72.0, *n* = 91), 23.4 ± 23.0 (0–83.0, *n* = 61), 21.6 ± 16.7 (0–51.0, *n* = 25), and 24.5 ± 19.7 (0–75.0, *n* = 37), respectively, and the mean relative change rates of BFMDRS-M were 57%, 67%, 57.8%, 50.15%, and 50.8% compared to the preoperative score, respectively (*p* ≤ 0.001 for all follow-up time categories). In conclusion, the BFMDRS motor scores were significantly improved by GPi-DBS compared to the preoperative scores at the last follow-up (49.3 ± 24.5 vs. 20.9 ± 21.4, *p* < 0.001).

Regarding the BFMDRS-D score (*n* = 84), GPi-DBS showed a significant improvement over baseline at the last follow-up (14.8 ± 7.0 vs. 7.0 ± 7.0, *p* < 0.001). The average BFMDRS-D scores for 0- to ≤6-, >6- to ≤12-, >12- to ≤24-, >24- to ≤36-, and >36-month time periods were 8.9 ± 8.5 (0–29, *n* = 33), 5.7 ± 6.4 (0–29, *n* = 51), 8.8 ± 8.2 (0–29, *n* = 24), 5.8 ± 5.4 (0–17, *n* = 12), and 7.4 ± 5.9 (0–20, *n* = 21), respectively, and the corresponding mean relative change rates of BFMDRS-D were 52.5%, 62.6%, 48.2%, 61.2%, and 55.2% compared to the preoperative scores, respectively (*p* ≤ 0.001 for all follow-up time categories).

### 3.5. Subgroup Analysis between Different Genotypes of Early-Onset Dystonia

The results showed that there were significant differences in the BFMDRS-M preoperative and postoperative scores between the DYT-6 (+) and DYT-11 (+) groups (49.5 vs. 23.9, *p* < 0.001, for preoperative scores and 24.7 vs. 7.0, *p* < 0.001, for postoperative scores, respectively), with significant differences in the mean improvement (52% vs. 68.7%, *p* = 0.008). The improvement rates in the BFMDRS-M score for the DYT-6 (+) and DYT-1 (+) groups were 52.0% and 71.8% (*p* = 0.001), respectively, and the postoperative BFMDRS-M scores were significantly different between the DYT-6 (+) and DYT-1 (+) groups (49.5 vs. 15.4, *p* = 0.027). However, there was no significant difference in the improvement rate of BFMDRS-M score between the DYT-1 (+) and DYT-11 (+) groups (68.7% vs. 71.8%, *p* = 0.545). With regard to the BFMDRS-D score, there was difference in the improvement rate between the DYT-6 (+) and DYT-11 (+) groups (46.7% vs. 78.3%, *p* = 0.010). Although no significant difference was found after comparison, the improvement rates of BFMDRS-D scores in the DYT-11 (+) and DYT-1 (+) groups were 78.3% and 63.1% (*p* = 0.086), respectively, and 78.3% and 46.7% (*p* = 0.230) in the DYT-1 (+) and DYT-6 (+) groups, respectively.

### 3.6. Follow-Up and Result with a Limited Follow-Up Time of 3 Years

A longer length of follow-up was considered ≥36 months. However, due to the different follow-up times for each genotype, we extracted data for a three-year follow-up period to remove significant heterogeneity among follow-up lengths. The average follow-up time after surgery was 12 months (range of 1–36 months). There was no difference in the results regarding the improvement rate. The postoperative BFMDRS-M scores of the DYT-6 (+) and DYT-1 (+) groups were not significantly different (22.1 vs. 16.3, *p* = 0.235), which was different from the undefined follow-up time.

### 3.7. Correlation and Metaregression Analysis

**Figure 2:**
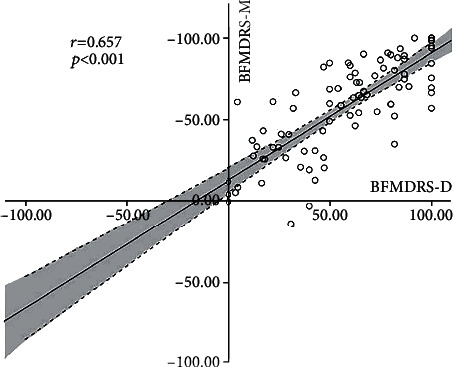
Correlation between relative improvement (%) in BFMDRS-M and BFMDRS-D at the last follow-up visit. There is a positive linear correlation between the relative improvement of BFMDRS-M and BFMDRS-D (Pearson *r* = 0.657, *p* < 0.001). Dots: individual patient values; black solid line: linear regression line; area between the dotted lines: 95% confidence interval; BFMDRS: Burke–Fahn–Marsden Dystonia Rating Scale; BFMDRS-D: BFMDRS disability subscale; BFMDRS-M: BFMDRS movement subscale.

**Figure 3:**
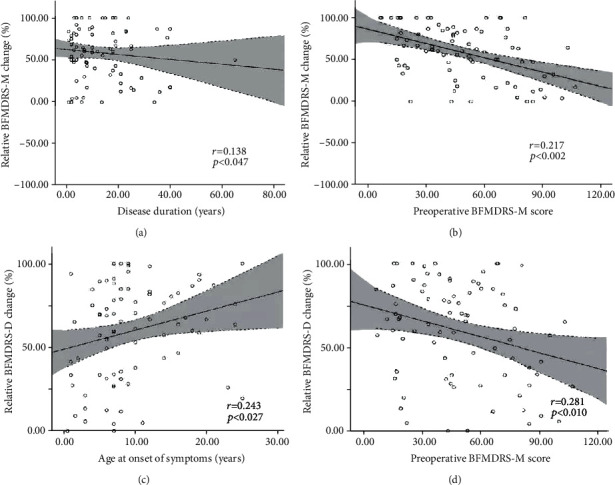
Potential predictive factors for relative improvement (%) in BFMDRS-M and BFMDRS-D under 3 years of follow-up visit. There were significant positive correlations between (a) disease duration and the relative improvement (%) in BFMDRS-M (Pearson *r* = −0.138, *p* = 0.047), (b) preoperative BFMDRS-M score (*r* = −0.217, *p* = 0.002) and the relative improvement (%) in BFMDRS-M, (c) age at onset (*r* = 0.243, *p* = 0.027) and the relative improvement (%) in BFMDRS-D, and (d) preoperative BFMDRS-M score (*r* = −0.281, *p* = 0.010) and the relative improvement (%) in BFMDRS-D. Dots: individual patient values; black solid line: linear regression line; area between the dotted lines: 95% confidence interval; BFMDRS: Burke–Fahn–Marsden Dystonia Rating Scale; BFMDRS-D: BFMDRS disability subscale; BFMDRS-M: BFMDRS movement subscale.

The relative changes of BFMDRS-D and BFMDRS-M after surgery had a good correlation (Pearson *r* = 0.657, *p* < 0.001; Figure 2). In general, the decrease in BFMDRS-M score was accompanied by a decrease in BFMDRS-D score. In addition, to determine outcome predictors, the relationship between various preoperative variables and the decrease of BFMDRS-M at 3 years after surgery was tested, or there was no follow-up restriction. Each demographical and clinical factor was tested separately. A summarized scatter plot showing the results of each factor is provided in Figure 3. The relative change rates in BFMDRS-M were negatively correlated with preoperative BFMDRS-M score (*r* = −0.217, *p* = 0.002) and disease duration (years) (*r* = −0.138, *p* = 0.047) during the 3-year follow-up period. In addition, the relative change rates in BFMDRS-D were positively correlated with preoperative BFMDRS-M score (*r* = −0.281, *p* = 0.010) and age at symptom onset (*r* = 0.243, *p* = 0.027) during the 3-year follow-up period. When no follow-up restrictions were set up, the relative change rates of BFMDRS-M were negatively correlated with the preoperative BFMDRS-M score (*r* = −0.205, *p* = 0.002). The relative change rates in BFMDRS-D were only positively correlated with age at symptom onset (*r* = 0.232, *p* = 0.027), whereas the association with age at surgery, course of disease, and follow-up period did not reach significance (Table 4).

Furthermore, stepwise multivariable regression analysis was performed to verify the possible predictors, and percent improvement (%) of BFMDRS-M was defined as dependent variable; age at onset, duration of surgery, duration of follow-up, and BFMDRS-M at baseline were included as independent variables. The best predictive model contained preoperative scores of BFMDRS-M (*β* = −0.280, *p* ≤ 0.001) and disease duration (*β* = −0.359, *p* = 0.014). As for BFMDRS-D score, independent variables were the same as BFMDRS-M and percent improvement (%) of BFMDRS-D was defined as dependent variable. The best predictive model contained only the preoperative scores of BFMDRS-M (*β* = −0.318, *p* = 0.010).

### 3.8. Complications

The adverse events are summarized in Table 5. Complications were described in 34 of 231 patients according to the published follow-up reports (4 DYT-6 (+) patients, 9 DYT-11 (+) patients, 11 DYT-1 (+) patients, and 10 unknown-genotype early-onset dystonia patients). During the follow-up period of the 34 patients who had complications, one died from suicide but had no depression, three had depression but had no suicidal tendency, seven had microinfection, 14 had a hardware infection, six had persistent dystonia or showed an improvement, and two had electrode displacement. With regard to the left mesa lateral cranium (by 4.5 cm) 11 months after surgery, two cases showed a lead fracture, and one case showed complex partial fractures. In the case of hardware failures, IPG switched off occurred in 9 patients. These complications generally occurred after one year. Most of the complications due to hardware failure occurred in the DYT-1 (+) group.

## 4. Discussion

We extracted all available data from various publications on patients with early-onset dystonia treated with DBS based on individual patient-level data and meta-analysis. We confirmed that GPi-DBS not only has a beneficial effect on motor and disability results in the treatment of early-onset dystonia but also has obtained a remarkable effect. Shorter disease course and lower BFMDRS-M score are related to a better outcome. Differential improvement was observed in DYT-1, DYT-6, DYT-11, and unknown genotype, and it was positively affected by a lower BFMDRS-M score (DYT-6 (+) effect was lower than DYT-1 (+)) and older age at symptom onset (DYT-11 (+), which was opposite of DYT-6 (+)). Our results suggested that the different genotypes of dystonia may contribute to the effect of disease duration on the outcome of GPi-DBS. For example, DYT-1 and DYT-11 carriers obtained more effective results compared to the other genotypes.

### 4.1. Target of DBS

The thalamic ventralis intermedius nucleus (Vim) is the initial described target of DBS, which is a valid treatment for refractory dystonia, but the GPi takes the place of it quickly [22]. Afterwards, the subthalamic nucleus (STN) was also proved to be a target structure for the treatment of dystonia [23]. As for early-onset dystonia, the STN DBS target was proved to be well tolerated with minor and transient adverse effects [24]. At the same time, in refractory generalized and segmental dystonia, GPi-DBS is a safe and reliable treatment option [25] and it is often applied to hereditary dystonia. Besides, the target of most of the published literature on the remedy of early-onset dystonia is GPi. Therefore, our study discussed the bilateral GPi-DBS treating early-onset dystonia.

### 4.2. Comparison of BFMDRS-M among Different Genotypes of Early-Onset Dystonia

For movement symptom severity, GPi-DBS was more effective in DYT-1 (+) and DYT-11 (+) patients compared to DYT-6 (+) patients. Moreover, the change rate of BFMDRS-M between DYT-1 (+) and DYT-11 (+) patients was not significantly different on account of many studies have confirmed that GPi-DBS has significant improvement and certain safety for DYT-1 (+) patients and on DYT-11 (+) syndrome [7, 13, 26–30]. Considering the treatment effect is affected by the time after surgery, we set a restriction of 3 years for follow-up time. After reanalysis, the data reprocessing showed the same results that strengthens our trust in the analysis results. The lack of significant difference in the postoperative BFMDRS-M scores may be due to the unpredictable effect of GPi-DBS on DYT-6 (+) patients compared to DYT-1 (+) and DYT-11 (+) patients [31]. Direct comparisons between DYT-11 (+) and DYT-1 (+) patients were hard to perform on account of some significant between-group differences in their demographic, baseline severity, and clinical factors found. However, no significant difference was found in outcomes among DYT-11 (+) and DYT-1 (+) patients, which was in accordance with previous study [13].

In addition, patients with 6 are more unpredictable. GPi-DBS makes significant outcomes one year for patients of all genotypes [26, 32]. The different degrees of exercise benefits observed in DYT-6 (+) and DYT-1 (+) patients may be due to the different patterns of metabolic abnormalities reported in these two monogenotypes in the dystonia connectivity study [13, 33, 34]. The effect was even more pronounced 12 months after surgery. As time goes on, there may be consequences of failure or need for intervention after a long period of operation, and even serious complications may occur. Carriers of DYT-6 mutation are more likely to undergo additional neurosurgery after surgery, suggesting that eligible patients with severe dystonia should be screened for the mutation before treatment with GPi-DBS. Patients diagnosed as DYT-6 mutation carriers who receive GPi-DBS should be informed of the failure of primary treatment and the possible risk of secondary intervention [31]. Therefore, some authorities recommend for testing DYT-1 and DYT-11, which has a favorable result, and others suggest testing for DYT-6 to inform patients that their surgical success rate may not be high. This may be a reference for patients and can be used as one of the factors influencing the effect of GPi-DBS treatment.

Our results suggested that different genotypes of dystonia may contribute to the effect of disease duration on the outcome of GPi-DBS treatment. Moreover, the outcome of GPi-DBS treatment for early-onset dystonia is related to genes as DYT-1 carriers obtained more effective results compared to other genotypes which is consistent with previous literature [13]. DYT-11 carriers also showed significant improvement in dystonia severity and disability outcomes. Therefore, genetic testing can improve the accuracy of diagnosis and enable patients to have a more accurate and detailed understanding of the expected results.

### 4.3. Comparison between Outcomes of BFMDRS-D among Different Genotypes of Early-Onset Dystonia

For disability symptom severity, outcomes of BFMDRS-D were the same regardless of follow-up time restriction for the limited data of BFMDRS-D. Some studies suggested that myoclonus is related to globus pallidus, but there is no strong evidence for the specific mechanism [35]. In view of the fact that GPi-DBS treatment of patients in DYT-11 (+) is capable of improving symptoms and treating myoclonus at the same time to achieve a good effect [30], the change rate of DYT-11 (+) patients was higher than that of DYT-6 (+) patients, which was consistent with the previous literature [2]. Further, considering many trials have shown that the GPi-DBS treatment is effective for DYT-1 (+) patients [36] and more patients in DYT-1 (+) patients, there was no significant difference in the GPi-DBS treatment effect between DYT-1 (+) and DYT-11 (+) patients. Mutated DYT-1 may cause abnormal neurotransmission and interfere with neuronal firing in brain motor pathways [37], and GPi-DBS may block the abnormal discharge pattern of GPi, thereby reducing the excessive activation of the cortex [38]. Moreover, there was no significant effect between DYT-1 (+) and DYT-6 (+) patients, which may be due to the different patterns of metabolic abnormalities reported in these two monogenotypes in the dystonia connectivity study as described before. Our study showed that there was a correlation between preoperative BFMDRS-M score and the relative improvement rate of BFMDRS-D, and the preoperative BFMDRS-M score between DYT-1 (+) and DYT-6 (+) patients had no significant difference. Thus, change rate in these comparisons had no significant difference.

### 4.4. Predictors

The present study demonstrated that BFMDRS-M and BFMDRS-D were positively correlated, indicating that GPi-DBS does not only improve symptoms but also reduce disability [17]. Many studies have investigated the potential predictive factors of DBS for early-onset dystonia. All data were included and analyzed in the present meta-analysis, revealing that patients with lower preoperative BFMDRS-M scores obtained higher improvement rates of the BFMDRS-M score. Furthermore, in an article studying DBS in patients with Meige syndrome, Wang et al. found higher efficacy was related to lower preoperative BFMDRS-M scores and provided a possible explanation in that patients with higher BFMDRS scores or multiple onset sites have a greater overall disease burden and more likely to have unsatisfactory clinical outcomes [39]. Further research is needed to confirm this finding. Later age of onset was a beneficial factor for BFMDRS-D because DYT-11 (+) patients with later age of onset benefit the most from GPi-DBS [13].

Regarding the impact of the follow-up period, we performed a second analysis after data were limited to a 3-year follow-up period. Our results were consistent with a recent metaregression analysis that reported an association between shorter duration of dystonia and greater improvement in motor score after GPi-DBS treatment of patients with isolated dystonia [18, 40]. One possible explanation is that longer disease duration may lead to a greater burden of bone deformities secondary to the disease dystonia [28]. However, people with skeletal deformities are least likely to get symptom relief from DBS [41]. Moreover, patients with lower score of preoperative BFMDRS-M obtained higher improvement rates of BFMDRS-M and BFMDRS-D. To explain the different results after a limited time, the effect may not be as good when the time is prolonged. One study has shown that operation at a younger age is related to the favorable improvement of symptoms in isolated dystonia [27] since younger patients have better tolerance. However, our results showed that patients with older age of onset achieve more beneficial results. In early-onset dystonia, the age of onset often indirectly leads to the shortening of the course of disease. In addition, the course of the disease was positively correlated with the BFMDRS-M score.

In general, shorter disease course and lower BFMDRS-M score are associated with a better outcome. Furthermore, univariate metaregression indicated a remarkable negative correlation between greater baseline damage and a greater effect of DBS (i.e., higher BFMDRS-M and BFMDRS-D). Therefore, when a disability does not respond to medicine and the quality of life declines, GPi-DBS surgery should be performed [29].

### 4.5. Complications

Compared to pallidotomy, the complications of DBS surgery involve the hardware [42]. Most of the complications occurred after a long follow-up period. In bilateral GPi-DBS, device infection remained the most significant adverse event with more hardware failures, less symptom aggravation, and only one patient died. The treatment of complications mentioned in the literature was also less. Because most of the complications were hardware-related complications, reoperation or electrode replacement was undergone to relieve symptoms caused by complications.

### 4.6. Mechanism of DBS for Early-Onset Dystonia

Deep brain stimulation (DBS) is a commonly used intervention to treat dystonia by neuromodulation, and DBS has a significant benefit in up to 90% of children with primary or hereditary dystonias [43]. Specific brain regions can be targeted to significantly improve motor performance. Studies suggested that DBS is well matched with the functional consequences of brain injury, and its impact is most likely due to the scale of downstream target input and the lack of information transmission, rather than the lack of information in a specific pattern [44].

Through direct comparison, it is concluded that the response of DYT-6 mutation patients is more difficult to predict than the response of DYT-1 mutation patients. Excluding genetic influence, some of this variability may be related to stimulus settings and/or lead position [45, 46]. GPi-DBS supports the concept of cortical regulation during high-frequency stimulation by improving the local effects of relative motion locations and inhibiting the consistency of electroencephalographic [47].

Thus, GPi-DBS can affect neuronal activity in local and other functional connections, and components of the cortices-basal ganglia neural network can cause long-term plasticity changes at the cortical level, which can then reestablish normal movement. This cortical modulation may be the explanation why improvements in tetanic motion may delay DBS by weeks and months [29].

Combined with local and distant modulation in neuronal networks, DBS may play an important role in the neurochemical system [48]. Under the premise of microdialysis or voltammetric analysis based on animal models, imaging studies or direct evaluation has involved DBS in the process of modulation of neurotransmitter release such as dopamine, glutamate, and gamma-aminobutyric acid; however, there is currently no specific research on dystonia [29].

### 4.7. Study Limitations

Several limitations of our study merit mention. First, the insufficiency of our meta-analysis is that the number of patients with DYT-1 (+) was more than twice that of patients with DYT-6 (+) and combined dystonia. Combined dystonia includes DYT-11 (+) and DYT-28 (+) patients, and DYT-28 is a new single-gene inherited early-onset dystonia discovered only in recent years. Thus, there are few relevant studies for combined dystonia.

Second, improvement on a dystonia motor scale does not automatically imply an improvement of quality of life as was previously shown in stimulated patients with generalized dystonia [49]. However, patients evaluated in specialized scales such as SF-36 were few due to the inclusion and exclusion criteria that we strictly followed, and the outcome in our study was only based on BFMDRS-M and BFMDRS-D. Thus, more scoring scales are needed in future studies to more comprehensively and quantitatively evaluate the health-related quality of life and mental status of patients.

Third, the study is based on an unblinded evaluation of published cases. Hence, our conclusions tend to deviate from the expectations of patients and physicians, as well as publication bias that tend to favor better outcomes. However, because we used a conservative approach, the results described may underestimate the actual DBS benefits.

To date, there is limited evidence for short-term benefits, and data for long-term results after treatment of early-onset dystonia with GPi-DBS are also limited. Based on the fact that DBS is an inherent surgical intervention, it should be evaluated and discussed by an experienced multidisciplinary dyskinesia team.

## 5. Conclusion

The motor and disability symptoms of early-onset dystonia with DYT-1 (+), DYT-6 (+), DYT-11 (+), and DYT-28 (+) genotypes are effectively improved by GPi-DBS. For movement symptom severity, DYT-1 (+) patients and DYT-11 (+) patients are potential candidates for better results with GPi-DBS clinically. For DYT-11 (+) patients with severe disability, GPi-DBS treatment is a great choice. Patients who have been treated with GPi-DBS need to be reviewed or even undergo another surgery after one year. Lower preoperative BFMDRS-M score and shorter disease course indicate better efficacy of movement symptom. Lower preoperative BFMDRS-M score and later age of onset indicate better efficacy of disability symptom. Consequently, GPi-DBS should be considered in a timely manner once the symptoms cannot be controlled by drugs.

## Figures and Tables

**Table 1:** Demographic characteristic, baseline dystonia severity, and programming parameters.

	All patients (*n* = 231)	DYT-6 (*n* = 27)	DYT-11 (*n* = 32)	DYT-1 (*n* = 101)	Unknown (*n* = 71)	DYT-6 and DYT-11	DYT-6 and DYT-1	DYT-11 and DYT-1
Male/female/unknown	110/98/26	16/11/0	15/15/2	43/50/8	36/22/13	0.263	0.075	0.640
Age at symptom onset (years), mean/median (range)	8.9/8 (0-25)	9.4/8 (3-25)	7.7/5.5 (0.5-25)	9.1/8 (1-24)	8.9/8 (0-25)	0.277	0.752	0.254
Age at surgery (years), mean/median (range)	24.3/19 (5.3-72)	22.8/20 (8-48)	29.5/27.5 (8-72)	21.5/17 (5.3-65.5)	26.4/22 (7-64)	0.065	0.626	**0.005**
Disease duration before surgery (years), mean/median (range)	15.4/11 (0.5-66)	13.4/11 (2-39)	21.7/18.3 (2-66)	12.4/7 (1-56.5)	17.5/12 (0.5-58)	**0.015**	0.670	**≤0.001**
Intraoperative microelectrode recording used (yes/no/not reported)	120/21/93	10/4/13	13/3/16	59/7/35	38/7/29	0.960	0.079	0.084
Baseline BFMDRS-M (/120), mean/median (range)	49.3/47.5 (4-130)	49.5/44 (16-89)	23.9/18.8 (4-75)	49.8/50 (16.5-107)	58.9/58 (11-130)	**≤0.001**	0.941	**≤0.001**
Postoperative BFMDRS-M (/120), mean/median (range)	20.9/14 (0-94.5)	24.7/18 (3-69.6)	7.0/5 (0-21.5)	15.4/9 (0-94.5)	33.5/33 (0-87)	**≤0.001**	**0.027**	**≤0.001**
Mean change rate (%) of BFMDRS-M (range)	60.6 (-33.8-100)	52.0 (-2.5-92.7)	68.7 (13.2-100)	71.8 (4.1-100)	44.4 (-33.8-100)	**0.008**	**0.001**	0.545
Baseline BFMDRS-D (/30), mean/median (range)	14.7/14 (3-30)	11.2/9.5 (3-21)	8.7/7.5 (5-18)	15.7/14.5 (6-29)	17.8/18 (7-30)	0.458	0.199	**≤0.001**
Postoperative BFMDRS-D (/30), mean/median (range)	6.9/4 (0-29)	6.2/4.5 (1-18)	1.8/2 (0-6)	6.1/4 (0-25)	11.3/10 (2-29)	0.150	0.976	**0.001**
Mean change rate (%) of BFMDRS-D (range)	57.5 (0-100)	46.7 (14.3-88.9)	78.3 (25-100)	63.1 (0-100)	38.9 (0-86.7)	**0.010**	0.230	0.086
Length of follow-up (months), median (range)	12 (1-196)	13 (2-196)	9 (1-20)	12 (1-102)	12 (5-50)	**0.003**	**0.010**	0.061
Mean DBS programming parameters±SD	*N* = 98	*N* = 9	*N* = 12	*N* = 42	*N* = 35			
Voltage(V) Rt	2.8 ± 1.1	2.9 ± 0.7	3.2 ± 0.9	2.8 ± 1.2	2.6 ± 1.3	0.498	0.700	0.326
Voltage(V) Lt	2.7 ± 1.1	2.9 ± 0.7	3.0 ± 0.9	2.8 ± 1.2	2.5 ± 1.1	0.819	0.659	0.493
PW (*μ*s) Rt	155.4 ± 128.6	143.3 ± 118.8	126.9 ± 116.2	212.1 ± 159.4	100.3 ± 37.8	0.755	0.161	0.052
PW (*μ*s) Lt	152.4 ± 128.8	143.3 ± 118.8	126.9 ± 116.2	207.9 ± 162.1	96.9 ± 27.3	0.755	0.188	0.065
Frequency (Hz) Rt	124.5 ± 27.9	128.6 ± 33.3	121.7 ± 32.4	118.2 ± 30.2	132 ± 20.1	0.639	0.363	0.732
Frequency (Hz) Lt	124.5 ± 27.9	128.6 ± 33.3	121.7 ± 32.4	118.2 ± 30.2	132 ± 20.1	0.639	0.363	0.732

BFMDRS-M: Burke–Fahn–Marsden Dystonia Rating Scale-movement component; BFMDRS-D: Burke–Fahn–Marsden Dystonia Rating Scale-disability component.

**Table 2:** Demographic characteristic and baseline dystonia severity in 3 y follow-up.

	All patients (*n* = 210)	DYT-6 (*n* = 20)	DYT-11 (*n* = 32)	DYT-1 (*n* = 91)	Unknown (*n* = 67)	DYT-6 and DYT-11	DYT-6 and DYT-1	DYT-11 and DYT-1
Male/female/unknown	105/79/26	13/7/0	15/15/2	42/41/8	35/16/16	0.163	0.086	0.850
Age at symptom onset (years), mean/median (range)	8.9/8 (0-25)	10.1/9 (3-25)	7.7/5.5 (0.5-25)	9.1/8 (1-24)	8.7/8 (0-25)	0.187	0.365	0.259
Age at surgery (years), mean/median (range)	23.8/18.8 (5.3-72)	25.2/23 (8-48)	29.5/27.5 (8-72)	20.8/17 (5.3-65.5)	24.8/18.4 (7-63)	0.284	0.159	**0.002**
Disease duration before surgery (years), mean/median (range)	14.9/10 (0.5-66)	15.1/12.5 (2-39)	21.7/18.3 (2-66)	11.6/7 (1-56.5)	16/9 (0.5-58)	0.087	0.220	**≤0.001**
Intraoperative microelectrode recording used (yes/no/not reported)	118/21/71	10/4/6	13/3/16	57/7/27	38/7/22	0.634	0.468	0.892
Baseline BFMDRS-M (/120), mean/median (range)	49.4/48 (4-130)	48/42.5 (16-89)	23.9/18.75 (4-75)	49.8/50 (16.5-107)	59.5/58 (11-130)	**≤0.001**	0.582	**≤0.001**
Postoperative BFMDRS-M (/120), mean/median (range)	21.1/13 (0-94.5)	22.1/16.25 (1-69.6)	6.8/4 (0-21.5)	16.3/10 (0-94.5)	34.2/33.5 (0-87)	**0.002**	0.235	**≤0.001**
Mean change rate (%) of BFMDRS-M (range)	60.9 (-33.8-100)	55.6 (19.5-97.6)	69.8 (13.2-100)	71.2 (4.1-100)	44.2 (-33.8-100)	**0.032**	**0.017**	0.790
Baseline BFMDRS-D (/30), mean/median (range)	14.8/14 (3-30)	11.4/9 (3-21)	8.7/7.5 (5-18)	15.7/14.5 (6-29)	17.9/18 (7-30)	0.507	0.202	**≤0.001**
Postoperative BFMDRS-D (/30), mean/median (range)	7.0/4 (0-29)	6.2/3 (1-18)	1.8/2 (0-6)	6.4/4.5 (0-25)	11.5/10 (2-29)	0.234	0.961	**≤0.001**
Mean change rate (%) of BFMDRS-D (range)	57.1 (0-100)	47.9 (14.3-88.9)	78.3 (25-100)	61.9 (0-100)	37.9 (0-86.7)	**0.023**	0.343	0.061
Length of follow-up (months), median (range)	12 (1-36)	6.5 (2-36)	9 (1-20)	12 (1-36)	9 (5-36)	0.557	0.363	0.056

BFMDRS-M: Burke–Fahn–Marsden Dystonia Rating Scale-movement component; BFMDRS-D: Burke–Fahn–Marsden Dystonia Rating Scale-disability component.

**Table 3:** Clinical outcomes in different time categories.

Time category (months)	Mean BFMDRS-M score	Improvement (%) in BFMDRS-M score	*p* value	Mean BFMDRS-D score	Improvement (%) in BFMDRS-D score	*p* value
>0 and ≤6	23.6 ± 24.9 (0-94.5)	57	**≤0.001**	8.9 ± 8.5 (0-29)	52.5	**≤0.001**
>6 and ≤12	17.1 ± 17.9 (0-72)	67	**≤0.001**	5.7 ± 6.4 (0-29)	62.6	**≤0.001**
>12 and ≤24	23.4 ± 23 (0-83)	57.8	**≤0.001**	8.8 ± 8.2 (0-29)	48.2	**≤0.001**
>24 and ≤36	21.6 ± 16.7 (0-51)	50.1	**≤0.001**	5.8 ± 5.4 (0-17)	61.2	**0.001**
>36	24.5 ± 19.7 (0-75)	50.8	**≤0.001**	7.4 ± 5.9 (0-20)	55.2	**≤0.001**

The improvement of BFMDRS‐M(D) = (Preoperative scores − Postoperative scores)/Preoperative scores.

**Table 4:** Relationship between several factors and relative improvement (%) in BFMDRS.

Patient number	Follow-up period in 3 years		At the last follow-up	
	Improvement (%) in BMFDRS-M (*n* = 209)	Improvement (%) in BMFDRS-D (*n* = 83)	Improvement (%) in BMFDRS-M (*n* = 231)	Improvement (%) in BMFDRS-D (*n* = 91)
Age at onset of symptoms (years)	*r* = 0.068	**r** = 0.243	*r* = 0.083	**r** = 0.232
	*p* = 0.327	**p** = 0.027	*p* = 0.208	**p** = 0.027

Age at surgery (years)	*r* = −0.102	*r* = 0.088	*r* = −0.082	*r* = 0.118
	*p* = 0.143	*p* = 0.427	*p* = 0.212	*p* = 0.264

Disease duration (years)	**r** = −0.138	*r* = −0.021	*r* = −0.123	*r* = 0.020
	**p** = 0.047	*p* = 0.851	*p* = 0.063	*p* = 0.854

Follow-up period (months)	*r* = −0.059	*r* = −0.048	*r* = −0.026	*r* = −0.075
	*p* = 0.396	*p* = 0.668	*p* = 0.697	*p* = 0.478

Preoperative BFMDRS-M score	**r** = −0.217	**r** = −0.281	**r** = −0.205	*r* = −0.192
	**p** = 0.002	**p** = 0.010	**p** = 0.002	*p* = 0.068

Disease duration refers to the time from the onset to the operative treatment.

**Table 5:** Frequency of complications following DBS for early-onset dystonia.

At the last follow-up	
Death	1
Surgical infection (electrode infection (*n* = 2), battery infection (*n* = 1), and device infection (*n* = 4))	7
Hardware failure (electrode (*n* = 1), electrode misplacement (*n* = 2), lead fracture (*n* = 2), and internal pulse generators switched off (*n* = 9))	14
Continued worsening of dystonia	6
Unimproved	2
Depression	3
Having complex partial seizures	1

## Data Availability

The initial data supporting this meta-analysis are from previously reported studies and datasets, which have been cited. The processed data are available (in the Supplementary Materials).

## Abbreviations

DBS: Deep brain stimulation
GPi: Globus pallidus internus
Vim: Ventral intermediate internus
STN: Subthalamic nucleus
BFMDRS: Burke–Fahn–Marsden Dystonia Rating Scale
BFMDRS-M: BFMDRS movement subscale
BFMDRS-D: BFMDRS disability subscale
IPDL: Individual patient data
MER: Microelectrode recording
IPG: Implanted pulse generator
OCEBM: Oxford Centre for Evidence-based Medicine.

## Supplementary Materials

Supplementary Materials Highlights demonstrate the main content and novelty of this article. Table S1 integrates and summarizes the original data and results of the 54 articles used in this meta-analysis.
