# Supplementary material for: The Efficacy and Predictors of Using GPi-DBS to Treat Early-Onset Dystonia: An Individual Patient Analysis
Source: Neural Plast. 2021 May 7;2021:9924639. doi: 10.1155/2021/9924639 (PMC8121596; doi:10.1155/2021/9924639)
Supplement: Supplementary Materials — Highlights demonstrate the main content and novelty of this article. Table S1 integrates and summarizes the original data and results of the 54 articles used in this meta-analysis. [file 9924639.f1.docx]

**Table S1.** Characteristics of the included 54 studies.

| Study | Number of patients | Including types | Mean age at onset, ±SD | Mean age at surgery, ±SD | Mean disease duration, ±SD | Mean months after surgery at the last FU, ±SD | Mean preoperative BFMDRS-M score, ±SD | Mean postoperative BFMDRS-M score, ±SD | Improvement (%) in BFMDRS-M score |
| --- | --- | --- | --- | --- | --- | --- | --- | --- | --- |
| Ben-Haim et al[1] | 5 | DYT1 | 7.2±0.8 | 11.6±2.6 | 4.4±3.0 | 21.2±45.2 | 64.2±38.3 | 34.7±41.8 | 51.8±42.6 |
| Borggraefe  et al [2] | 6 | DYT1 and unknown | 9±2.4 | 14.3±3.7 | 5.3±4.5 | 14.8±3.5 | 51.2±15.4 | 14±12.8 | 74.9±18.3 |
| Cersosimo et al[3] | 7 | DYT1 | 10.7±6.9 | 14.6±6.8 | 3.9±1.2 | 12±0 | 47.6±21.5 | 16.3±12.3 | 63.9±20.6 |
| Goto et al[4] | 3 | DYT1 | 9.3±2.5 | 21±6 | 11.7±4.9 | 12±0 | 53.5±19.8 | 1.2±1.6 | 97.8±2.5 |
| Lin et al[5] | 1 | DYT1 | 3 | 5.3 | 2.3 | 6 | 100 | 94.5 | 5.5 |
| Markun et al[6] | 11 | DYT1 | 7.6±2.1 | 15.9±5.9 | 8.3±7.1 | 30.4±19.3 | 53.5±22.7 | 19.5±18.8 | 66.9±25 |
| Petrossian et al[7] | 10 | DYT1 and unknown | 9.9±3 | 15.5±4 | 5.6±3.9 | 6±0 | 41.1±25.2 | 27.2±26.7 | 36.5±39.2 |
| Starr et al[8] | 19 | DYT1 and unknown | 9.5±6.4 | 33.8±16.3 | 24.3±16.9 | 17.4±8.7 | 47.3±22 | 25.9±21 | 56.1±33.4 |
| Tronnier et al [9] | 3 | DYT1 and unknown | 10±2 | 24.3±5.5 | 14.3±5.7 | 6±0 | 79.3±4.0 | 45.2±9.2 | 42.6±14.1 |
| Vidailhet et al[10] | 7 | DYT1 | 7.6±1.4 | 28.9±7.6 | 21.3±7.7 | 12±0 | 55.1±21.9 | 26.1±16.5 | 54.6±29.5 |
| Tisch et al[11] | 1 | DYT1 | 9 | 16 | 7 | 6 | 20 | 1 | 95 |
| Azoulay et al[12] | 5 | DYT11 | 14.6±5.6 | 44.6±15.7 | 30±19.8 | 15±0 | 33.1±13.3 | 6.5±5.8 | 80.7±12 |
| Candela et al[13] | 5 | DYT11 and unknown | 5.3±3 | 12.2±4 | 6.9±4.7 | 6±0 | 39.7±25 | 12.5±9.2 | 66.2±13.8 |
| Cif et al[14] | 1 | DYT11 | 1 | 8 | 7 | 20 | 18.5 | 2.5 | 86 |
| Fernandez  et al[15] | 3 | DYT11 | 6.3±9.3 | 32.7±12.1 | 26.1±14.4 | 7±4.6 | 43±29.5 | 6.3±8.5 | 87.0±11.4 |
| Foncke et al[16] | 2 | DYT11 | 5±2.8 | 28.5±14.8 | 23.5±17.7 | 6±0 | 16±8.5 | 6.5±3.5 | 59.6±0.6 |
| Kosutzka et al[17] | 9 | DYT11 | 9.1±7.3 | 32.7±16.6 | 23.6±16.2 | 8.4±2.8 | 17.4±13 | 4.3±5.5 | 71.4±28.3 |
| Kuhn et al[18] | 1 | DYT11 | 7 | 17 | 10 | 9 | 26 | 10.5 | 59.6 |
| Papuc et al[19] | 1 | DYT11 | 4 | 31 | 27 | 6 | 37 | 14 | 62.2 |
| Rocha et al[20] | 1 | DYT11 | 18 | 20 | 2 | 12 | 15 | 6 | 86.7 |
| Ruiz et al[21] | 1 | DYT11 | 2 | 26 | 24 | 12 | 12 | 6 | 50 |
| Uruha et al[22] | 1 | DYT11 | 5 | 42 | 37 | 2 | 14 | 4 | 71.4 |
| Bruggeman et al[23] | 5 | DYT6 | 10±4.3 | 18±8 | 8±6 | 75.4±60.2 | 55.6±17.5 | 33.2±18.9 | 42.4±19.6 |
| Chen et al[24] | 1 | DYT6 | 8 | 12 | 4 | 15 | 41 | 3 | 92.7 |
| Danielsson et al[25] | 7 | DYT6 | 7.1±2 | 16.9±6.6 | 9.7±5.5 | 79±75.8 | 57.8±20.4 | 27.5±21.6 | 56.9±19.4 |
| Franca et al[26] | 1 | DYT6 | 6 | 36 | 30 | 36 | 37 | 6 | 83.8 |
| Groen et al[27] | 4 | DYT6 | 8±1.8 | 29.8±17.3 | 21.8±15.9 | 6±0 | 40.9±27.2 | 25.5±16.4 | 34.3±15.6 |
| Panov et al[28] | 3 | DYT6 | 11.3±11.9 | 24.7±10.6 | 13.3±3.2 | 34±15.1 | 46±29 | 30±12.4 | 25±30.7 |
| Park et al[29] | 1 | DYT6 | 15 | 27 | 12 | 2 | 16 | 3 | 81.3 |
| Vuletic et al[30] | 1 | DYT6 | 7 | 16 | 9 | 12 | 52 | 4.5 | 91.3 |
| Kawarai et al[31] | 1 | DYT28  Excluded for lack of data | 7 | 17 | 9 | 1 | 35 | 19 | 45.7 |
| Kimura et al[32] | 3 | DYT6 | 12.3±6.8 | 31.3±10.6 | 19±8.2 | 12±0 | 50.3±35.9 | 26±28.8 | 57±20.2 |
| Mure et al[33] | 1 | DYT6 | 15 | 30 | 15 | 3 | 55 | 21 | 61.8 |
| Lumsden et al[34] | 2 | DYT11 | 3±0.1 | 11.3±2.2 | 7.6±3.4 | 9±4.2 | 31.5±2.1 | 15.5±8.5 | 51.6±23.7 |
| Kimura et al[35] | 1 | DYT11 | 2 | 17 | 15 | 12 | 21.5 | 16 | 25..6 |
| Kim et al[36] | 1 | DYT11 | 5 | 42 | 37 | 2 | 13.3 | 4 | 70 |
| Blomstedt et al[37] | 2 | DYT1 and unknown | 20.5±6.4 | 44.5±13.4 | 23.5±6.4 | 24±17 | 62±14.8 | 13.5±7.1 | 79±6.4 |
| Cheung et al[38] | 2 | DYT1 and unknown | 11±0 | 16.5±2.1 | 5.5±2.1 | 6±0 | 65±1.4 | 1.75±1.1 | 97.3±1.7 |
| Cif et al[39] | 11 | DYT1 | 8.7±3.9 | 20.3  ±17 | 11.5±16.6 | 12±0 | 56.8±19 | 5.9±7.3 | 86.4±19.6 |
| Jahanshahi et al[40] | 12 | DYT1 and unknown | 11.4±3.4 | 38.3±17.5 | 26.9±14.9 | 14.4±0 | 39.3±16.7 | 12.4±9.5 | 68.8±19.3 |
| Krause,M et al[41] | 11 | DYT1 and unknown | 8.8±6.2 | 25.8±9.0 | 17±7.2 | 6.2±0.9 | 81.0±12 | 52.0±25.1 | 36.3±29.2 |
| Krause,P et al[42] | 8 | DYT1 and unknown | 7.0±2.4 | 12.5±3.5 | 5.5±3.5 | 12±0 | 45.4±21.2 | 17.6±7.6 | 54.4±25.3 |
| Ruge et al[43] | 5 | DYT1 and unknown | 10±5.7 | 18.6±11.1 | 8.6±10.5 | 8±2.9 | 59.8±16.5 | 12±8.2 | 78.2±15.9 |
| Schrader et al[44] | 1 | DYT1 | 12 | 48 | 36 | 26 | 33 | 12 | 63.6 |
| Sobstyl et al [45] | 1 | DYT1 | 14 | 17 | 3 | 24 | 25 | 5 | 80 |
| Starr et al[46] | 4 | DYT1 | 7±0.8 | 9.5±1.9 | 2.5±1.3 | 12±0 | 42.1±20 | 3.3±4.3 | 86.5±21.4 |
| Tisch et al[47] | 5 | DYT1 | 10.6±3.4 | 34.6±15.5 | 24±13.1 | 6±0 | 34.5±15.9 | 8.7±9.3 | 79.8±16.6 |
| Tisch et al[48] | 7 | DYT1 | 10.6±2.9 | 33.3±14.6 | 22.7±12.5 | 6±0 | 35.4±14.3 | 7±7.6 | 82.6±14.4 |
| Finger et al[49] | 4 | DYT1 and unknown | 14.5±8.9 | 45.8±20.6 | 31.3±14.4 | 6±0 | 48.3±12.9 | 13.3±14 | 71.9±30.6 |
| Coenen et al[50] | 1 | Unknown | 2 | 14 | 12 | 24 | 54 | 9.5 | 82.4 |
| Lenders et al[51] | 1 | Unknown | 18 | 19 | 1 | 8 | 90 | 68 | 24.4 |
| Parr et al[52] | 4 | DYT1 and unknown | 5.2±4.5 | 11.5±3.5 | 6.3±1.6 | 6±0 | 82.8±15.6 | 34.5±21.4 | 59.5±24.1 |
| Sensi et al[53] | 5 | DYT1 and unknown | 14.8±3.3 | 31.2±7 | 16.4±5.3 | 36±0 | 45.9±8.3 | 15.3±9.4 | 68.1±14.9 |
| Zorzi et al[54] | 12 | DYT1 and unknown | 4.6±4 | 15.2±7.2 | 10.5±6.5 | 20±13 | 65.3±18.4 | 41.6±22.7 | 37.3±30.1 |

1. Ben-Haim S, Flatow V, Cheung T, Cho C, Tagliati M, Alterman RL: **Deep Brain Stimulation for Status Dystonicus: A Case Series and Review of the Literature**. *Stereotact Funct Neurosurg* 2016, **94**(4):207-215.

2. Borggraefe I, Mehrkens JH, Telegravciska M, Berweck S, Botzel K, Heinen F: **Bilateral pallidal stimulation in children and adolescents with primary generalized dystonia--report of six patients and literature-based analysis of predictive outcomes variables**. *Brain Dev* 2010, **32**(3):223-228.

3. Cersosimo MG, Raina GB, Piedimonte F, Antico J, Graff P, Micheli FE: **Pallidal surgery for the treatment of primary generalized dystonia: long-term follow-up**. *Clin Neurol Neurosurg* 2008, **110**(2):145-150.

4. Goto S, Yamada K, Shimazu H, Murase N, Matsuzaki K, Tamura T, Nagahiro S, Kuratsu J, Kaji R: **Impact of bilateral pallidal stimulation on DYT1-generalized dystonia in Japanese patients**. *Mov Disord* 2006, **21**(10):1785-1787.

5. Lin JP, Kaminska M, Perides S, Gimeno H, Baker L, Lumsden DE, Britz A, Driver S, Fitzgerald-O'Connor A, Selway R: **Bilateral globus pallidus internus deep brain stimulation for dyskinetic cerebral palsy supports success of cochlear implantation in a 5-year old ex-24 week preterm twin with absent cerebellar hemispheres**. *Eur J Paediatr Neurol* 2017, **21**(1):202-213.

6. Markun LC, Starr PA, Air EL, Marks WJ, Jr., Volz MM, Ostrem JL: **Shorter disease duration correlates with improved long-term deep brain stimulation outcomes in young-onset DYT1 dystonia**. *Neurosurgery* 2012, **71**(2):325-330.

7. Petrossian MT, Paul LR, Multhaupt-Buell TJ, Eckhardt C, Hayes MT, Duhaime AC, Eskandar EN, Sharma N: **Pallidal deep brain stimulation for dystonia: a case series**. *J Neurosurg Pediatr* 2013, **12**(6):582-587.

8. Starr PA, Turner RS, Rau G, Lindsey N, Heath S, Volz M, Ostrem JL, Marks WJ, Jr.: **Microelectrode-guided implantation of deep brain stimulators into the globus pallidus internus for dystonia: techniques, electrode locations, and outcomes**. *J Neurosurg* 2006, **104**(4):488-501.

9. Tronnier VM, Fogel W: **Pallidal stimulation for generalized dystonia. Report of three cases**. *J Neurosurg* 2000, **92**(3):453-456.

10. Vidailhet M, Vercueil L, Houeto JL, Krystkowiak P, Benabid AL, Cornu P, Lagrange C, Tezenas du Montcel S, Dormont D, Grand S *et al*: **Bilateral deep-brain stimulation of the globus pallidus in primary generalized dystonia**. *N Engl J Med* 2005, **352**(5):459-467.

11. Tisch S, Rothwell JC, Limousin P, Hariz MI, Corcos DM: **The physiological effects of pallidal deep brain stimulation in dystonia**. *IEEE Trans Neural Syst Rehabil Eng* 2007, **15**(2):166-172.

12. Azoulay-Zyss J, Roze E, Welter ML, Navarro S, Yelnik J, Clot F, Bardinet E, Karachi C, Dormont D, Galanaud D *et al*: **Bilateral deep brain stimulation of the pallidum for myoclonus-dystonia due to epsilon-sarcoglycan mutations: a pilot study**. *Arch Neurol* 2011, **68**(1):94-98.

13. Candela S, Vanegas MI, Darling A, Ortigoza-Escobar JD, Alamar M, Muchart J, Climent A, Ferrer E, Rumia J, Perez-Duenas B: **Frameless robot-assisted pallidal deep brain stimulation surgery in pediatric patients with movement disorders: precision and short-term clinical results**. *J Neurosurg Pediatr* 2018, **22**(4):416-425.

14. Cif L, Valente EM, Hemm S, Coubes C, Vayssiere N, Serrat S, Di Giorgio A, Coubes P: **Deep brain stimulation in myoclonus-dystonia syndrome**. *Mov Disord* 2004, **19**(6):724-727.

15. Fernandez-Pajarin G, Sesar A, Relova JL, Ares B, Jimenez-Martin I, Blanco-Arias P, Gelabert-Gonzalez M, Castro A: **Bilateral pallidal deep brain stimulation in myoclonus-dystonia: our experience in three cases and their follow-up**. *Acta Neurochir (Wien)* 2016, **158**(10):2023-2028.

16. Foncke EM, Bour LJ, Speelman JD, Koelman JH, Tijssen MA: **Local field potentials and oscillatory activity of the internal globus pallidus in myoclonus-dystonia**. *Mov Disord* 2007, **22**(3):369-376.

17. Kosutzka Z, Tisch S, Bonnet C, Ruiz M, Hainque E, Welter ML, Viallet F, Karachi C, Navarro S, Jahanshahi M *et al*: **Long-term GPi-DBS improves motor features in myoclonus-dystonia and enhances social adjustment**. *Mov Disord* 2019, **34**(1):87-94.

18. Kuhn AA, Krause P, Lauritsch K, Zentner C, Brucke C, Schneider GH: **Early surgical treatment in a case of myoclonus dystonia syndrome**. *J Child Neurol* 2014, **29**(11):NP149-150.

19. Papuc E, Obszanska K, Rejdak K, Stelmasiak Z, Trojanowski T: **Atypical symptomatology of myoclonus dystonia (DYT-11) with positive response to bilateral pallidal deep brain stimulation**. *Mov Disord* 2014, **29**(7):E3.

20. Rocha H, Linhares P, Chamadoira C, Rosas MJ, Vaz R: **Early deep brain stimulation in patients with myoclonus-dystonia syndrome**. *J Clin Neurosci* 2016, **27**:17-21.

21. Ruiz PJ, Ayerbe J, Bader B, Danek A, Sainz MJ, Cabo I, Frech FA: **Deep brain stimulation in chorea acanthocytosis**. *Mov Disord* 2009, **24**(10):1546-1547.

22. Uruha A, Kimura K, Okiyama R: **An Asian Patient with Myoclonus-Dystonia (DYT11) Responsive to Deep Brain Stimulation of the Globus Pallidus Internus**. *Case Rep Neurol Med* 2014, **2014**:937095.

23. Bruggemann N, Kuhn A, Schneider SA, Kamm C, Wolters A, Krause P, Moro E, Steigerwald F, Wittstock M, Tronnier V *et al*: **Short- and long-term outcome of chronic pallidal neurostimulation in monogenic isolated dystonia**. *Neurology* 2015, **84**(9):895-903.

24. Chen C, Cole W, Bronte-Stewart HM: **Hybrid cars may interfere with implanted deep brain stimulators**. *Mov Disord* 2009, **24**(15):2290-2291.

25. Danielsson A, Carecchio M, Cif L, Koy A, Lin JP, Solders G, Romito L, Lohmann K, Garavaglia B, Reale C *et al*: **Pallidal Deep Brain Stimulation in DYT6 Dystonia: Clinical Outcome and Predictive Factors for Motor Improvement**. *J Clin Med* 2019, **8**(12).

26. Franca S, Massano J, Linhares P, Rosas MJ, Volkmann J: **Pallidal Deep Brain Stimulation in DYT6: Significant Long-Term Improvement of Dystonia and Disability**. *Mov Disord Clin Pract* 2014, **1**(2):118-120.

27. Groen JL, Ritz K, Contarino MF, van de Warrenburg BP, Aramideh M, Foncke EM, van Hilten JJ, Schuurman PR, Speelman JD, Koelman JH *et al*: **DYT6 dystonia: Mutation screening, phenotype, and response to deep brain stimulation**. *Movement Disorders* 2010, **25**(14):2420-2427.

28. Panov F, Tagliati M, Ozelius LJ, Fuchs T, Gologorsky Y, Cheung T, Avshalumov M, Bressman SB, Saunders-Pullman R, Weisz D *et al*: **Pallidal deep brain stimulation for DYT6 dystonia**. *J Neurol Neurosurg Psychiatry* 2012, **83**(2):182-187.

29. Park JE, Vanegas-Arroyave N, Hallett M, Lungu C: **A Woman With a Novel Mutation of THAP1 With a Prominent Response to Deep Brain Stimulation of the Globus Pallidus Internus**. *JAMA Neurol* 2015, **72**(11):1369.

30. Vuletic V, Chudy D, Almahariq F, Dobricic V, Kostic V, Bogdanovic N: **Excellent outcome of pallidal deep brain stimulation in DYT6 dystonia: A case report**. *J Neurol Sci* 2016, **366**:18-19.

31. Kawarai T, Miyamoto R, Nakagawa E, Koichihara R, Sakamoto T, Mure H, Morigaki R, Koizumi H, Oki R, Montecchiani C *et al*: **Phenotype variability and allelic heterogeneity in KMT2B-Associated disease**. *Parkinsonism & Related Disorders* 2018, **52**:55-61.

32. Krause P, Brüggemann N, Völzmann S, Horn A, Kupsch A, Schneider GH, Lohmann K, Kühn A: **Long-term effect on dystonia after pallidal deep brain stimulation (DBS) in three members of a family with a THAP1 mutation**. *Journal of Neurology* 2015, **262**(12):2739-2744.

33. Mure H, Morigaki R, Koizumi H, Okita S, Kawarai T, Miyamoto R, Kaji R, Nagahiro S, Goto S: **Deep brain stimulation of the thalamic ventral lateral anterior nucleus for DYT6 dystonia**. *Stereotactic and Functional Neurosurgery* 2014, **92**(6):393-396.

34. Lumsden DE, Kaminska M, Gimeno H, Tustin K, Baker L, Perides S, Ashkan K, Selway R, Lin JP: **Proportion of life lived with dystonia inversely correlates with response to pallidal deep brain stimulation in both primary and secondary childhood dystonia**. *Dev Med Child Neurol* 2013, **55**(6):567-574.

35. Kimura Y, Mihara M, Kawarai T, Kishima H, Sakai N, Takahashi MP, Mochizuki H: **Efficacy of deep brain stimulation in an adolescent patient with DYT11 myoclonus-dystonia**. *Neurology and Clinical Neuroscience* 2014, **2**(2):57-59.

36. Ji Hee Kim MYCN, MD; Won Hee Lee, MD; Won Seok Chang, MD; Hyun Ho Jung, MD; Jin Woo Chang, MD, PhD: **Bilateral Globus Pallidus Interna Deep-Brain Stimulation in a Patient With Myoclonus-Dystonia: A Case Report**.

37. Blomstedt P, Hariz MI, Tisch S, Holmberg M, Bergenheim TA, Forsgren L: **A family with a hereditary form of torsion dystonia from northern Sweden treated with bilateral pallidal deep brain stimulation**. *Movement Disorders* 2009, **24**(16):2415-2419.

38. Cheung T, Zhang C, Rudolph J, Alterman RL, Tagliati M: **Sustained relief of generalized dystonia despite prolonged interruption of deep brain stimulation**. *Movement Disorders* 2013, **28**(10):1431-1434.

39. Cif L, Ruge D, Gonzalez V, Limousin P, Vasques X, Hariz MI, Rothwell J, Coubes P: **The influence of deep brain stimulation intensity and duration on symptoms evolution in an off stimulation dystonia study**. *Brain Stimulation* 2013, **6**(4):500-505.

40. Jahanshahi M, Torkamani M, Beigi M, Wilkinson L, Page D, Madeley L, Bhatia K, Hariz M, Zrinzo L, Limousin P *et al*: **Pallidal stimulation for primary generalised dystonia: Effect on cognition, mood and quality of life**. *Journal of Neurology* 2014, **261**(1):164-173.

41. Krause M, Fogel W, Kloss M, Rasche D, Volkmann J, Tronnier V: **Pallidal stimulation for dystonia**. *Neurosurgery* 2004, **55**(6):1361-1368.

42. Krause P, Lauritsch K, Lipp A, Horn A, Weschke B, Kupsch A, Kiening KL, Schneider GH, Kühn AA: **Long-term results of deep brain stimulation in a cohort of eight children with isolated dystonia**. *Journal of Neurology* 2016, **263**(11):2319-2326.

43. Ruge D, Cif L, Limousin P, Gonzalez V, Vasques X, Hariz MI, Coubes P, Rothwell JC: **Shaping reversibility? Long-term deep brain stimulation in dystonia: The relationship between effects on electrophysiology and clinical symptoms**. *Brain* 2011, **134**(7):2106-2115.

44. Schrader C, Capelle HH, Kinfe TM, Blahak C, Bäzner H, Lütjens G, Dressler D, Krauss JK: **GPi-DBS may induce a hypokinetic gait disorder with freezing of gait in patients with dystonia**. *Neurology* 2011, **77**(5):483-488.

45. Sobstyl M, Zabek M, Dzierzecki S, Mossakowski Z, Szczałuba K: **Successful bilateral pallidal stimulation in a patient with isolated lower limb dystonia coexistent with langerhans cell histiocytosis and coeliac disease**. *Neurologia i Neurochirurgia Polska* 2011, **45**(5):514-519.

46. Starr PA, Markun LC, Larson PS, Volz MM, Martin AJ, Ostrem JL: **Interventional MRI-guided deep brain stimulation in pediatric dystonia: First experience with the clearpoint system**. *Journal of Neurosurgery: Pediatrics* 2014, **14**(4):400-408.

47. Tisch S, Rothwell JC, Bhatia KP, Quinn N, Zrinzo L, Jahanshahi M, Ashkan K, Hariz M, Limousin P: **Pallidal stimulation modifies after-effects of paired associative stimulation on motor cortex excitability in primary generalised dystonia**. *Experimental Neurology* 2007, **206**(1):80-85.

48. Tisch S, Zrinzo L, Limousin P, Bhatia KP, Quinn N, Ashkan K, Hariz M: **Effect of electrode contact location on clinical efficacy of pallidal deep brain stimulation in primary generalised dystonia**. *Journal of Neurology, Neurosurgery and Psychiatry* 2007, **78**(12):1314-1319.

49. Finger ME, Siddiqui MS, Morris AK, Ruckart KW, Wright SC, Haq IU, Madden LL: **Auditory-Perceptual Evaluation of Deep Brain Stimulation on Voice and Speech in Patients With Dystonia**. *Journal of Voice* 2019.

50. Coenen VA, Rijntjes M, Sajonz B, Piroth T, Prokop T, Jost W, Trippel M, Urbach H, Reinacher PC: **Bilateral Globus Pallidus Internus Deep Brain Stimulation in a Case of Progressive Dystonia in Mohr-Tranebjaerg Syndrome with Bilateral Cochlear Implants**. *J Neurol Surg A Cent Eur Neurosurg* 2019, **80**(1):44-48.

51. Lenders MW, Vergouwen MD, Hageman G, van der Hoek JA, Ippel EF, Jansen Steur EN, Buschman HP, Hariz M: **Two cases of autosomal recessive generalized dystonia in childhood: 5 year follow-up and bilateral globus pallidus stimulation results**. *Eur J Paediatr Neurol* 2006, **10**(1):5-9.

52. Parr JR, Green AL, Joint C, Andrew M, Gregory RP, Scott RB, McShane MA, Aziz TZ: **Deep brain stimulation in childhood: an effective treatment for early onset idiopathic generalised dystonia**. *Arch Dis Child* 2007, **92**(8):708-711.

53. Sensi M, Cavallo MA, Quatrale R, Sarubbo S, Biguzzi S, Lettieri C, Capone JG, Tugnoli V, Tola MR, Eleopra R: **Pallidal stimulation for segmental dystonia: long term follow up of 11 consecutive patients**. *Mov Disord* 2009, **24**(12):1829-1835.

54. Zorzi G, Marras C, Nardocci N, Franzini A, Chiapparini L, Maccagnano E, Angelini L, Caldiroli D, Broggi G: **Stimulation of the globus pallidus internus for childhood-onset dystonia**. *Mov Disord* 2005, **20**(9):1194-1200.
